# Negative pressure therapy (vacuum) for wound bed preparation among diabetic patients: case series

**DOI:** 10.1590/S1516-31802009000300010

**Published:** 2009-10-06

**Authors:** Marcus Castro Ferreira, Viviane Fernandes de Carvalho, Fábio Kamamoto, Paulo Tuma, André Oliveira Paggiaro

**Affiliations:** 1 MD, PhD. Full professor and chairman, Plastic Surgery Division, Faculdade de Medicina da Universidade de São Paulo (FMUSP), São Paulo, Brazil.; 2 PhD. Attending physician at Hospital das Clínicas, Faculdade de Medicina da Universidade de São Paulo (FMUSP), São Paulo, Brazil.; 3 MD, MSc. Attending physician at Hospital Universitário, Faculdade de Medicina da Universidade de São Paulo (HC/FMUSP), São Paulo, Brazil.; 4 MD, PhD. Attending plastic surgeon at Hospital das Clínicas, Faculdade de Medicina da Universidade de São Paulo (HC/FMUSP), São Paulo, Brazil.; 5 MD. Medical preceptor, Plastic Surgery Division, Faculdade de Medicina da Universidade de São Paulo (FMUSP), São Paulo, Brazil.

**Keywords:** Diabetic foot, Skin transplantation, Surgical flaps, Negative-pressure wound therapy, Wound healing., Pé diabético, Transplante de pele, Retalhos cirúrgicos, Tratamento de ferimentos com pressão negativa, Cicatrização de feridas.

## Abstract

**CONTEXT:**

Complications from diabetes mellitus affecting the lower limbs occur in 40 to 70% of such patients. Neuropathy is the main cause of ulceration and may be associated with vascular impairment. The wound evolves with necrosis and infection, and if not properly treated, amputation may be the end result. Surgical treatment is preferred in complex wounds without spontaneous healing. After debridement of the necrotic tissue, the wound bed needs to be prepared to receive a transplant of either a graft or a flap. Dressings can be used to prepare the wound bed, but this usually leads to longer duration of hospitalization. Negative pressure using a vacuum system has been proposed for speeding up the treatment. This paper had the objective of analyzing the effects of this therapy on wound bed preparation among diabetic patients.

**CASE SERIES:**

Eighty-four diabetic patients with wounds in their lower limbs were studied. A commercially available vacuum system was used for all patients after adequate debridement of necrotic tissues. For 65 patients, skin grafts completed the treatment and for the other 19, skin flaps were used. Wound bed preparation was achieved over an average time of 7.51 days for 65 patients and 10 days for 12 patients, and in only one case was not achieved.

**CONCLUSIONS:**

This experience suggests that negative pressure therapy may have an important role in wound bed preparation and as part of the treatment for wounds in the lower limbs of diabetic patients.

## RESUMO

**CONTEXTO:**

Complicações do diabetes *mellitus* que afetam os membros inferiores ocorrem em 40 a 70% dos pacientes. A neuropatia é a principal causa de ulceração e pode estar associada com problemas vasculares. A ferida evolui com necrose e infecção, e se não for corretamente tratada poderá terminar em amputação. O tratamento cirúrgico é preferido em feridas complexas, quando não há cicatrização espontânea. Após desbridamento cirúrgico do tecido necrótico do leito da ferida este precisa ser preparado para receber um transplante, seja um enxerto ou um retalho. Curativos podem ser usados para o preparo do leito da ferida, mas frequentemente levam a um longo tempo de hospitalização. A pressão negativa usada através de um sistema vácuo foi proposta para acelerar o tempo de tratamento. O presente trabalho teve como finalidade analisar os efeitos desta terapia no preparo do leito de feridas em pacientes diabéticos.

**SÉRIE DE CASOS:**

Oitenta e quatro pacientes diabéticos com feridas em membros inferiores foram estudados. Um sistema vácuo de uso comercial foi utilizado em todos os pacientes após adequado desbridamento de tecidos necróticos. Em 65 pacientes enxertos de pele completaram o tratamento e em outros 19 retalhos cutâneos. O preparo do leito da ferida foi conseguido, em média, em 7,51 dias em 65 pacientes, em 10 dias para 12 pacientes e em somente um caso não foi efetivo.

**CONCLUSÃO:**

A experiência sugere que a terapia por pressão negativa possa ter um papel importante no preparo do leito e como parte do tratamento de feridas nos membros inferiores de pacientes diabéticos.

## INTRODUCTION

Diabetes mellitus is a chronic multifactorial disease. The worldwide prevalence was 120 million cases in 1996 but the prediction for 2025 is for 250 million cases, because of longer life expectancy, greater obesity and greater sedentarism.^1^ In Brazil, it has been estimated that around 10 million people are diabetic.^2^

The complications from this disease are serious and those affecting the lower limbs represent 40% to 70% of these patients.^3^ Such patients usually present lesions on their lower limbs that do not heal primarily. Although simply controlling blood glucose levels is important, this is not necessarily followed by healing of these ulcers.^4^ The wounds may evolve with extensive necrosis and infection, which may lead to amputation of parts of or even the whole limb.^5^^,^^6^

Obstruction of major blood vessels is responsible for less than 20% of these wounds.^7^ The main cause recognized today is neuropathy, consisting of progressive degeneration of sensitive nerves in the foot, induced by microangiopathy of the small vessels to the nerve fascicles. This is sometimes associated with external compression at anatomical sites, such as the tarsal tunnel.^8^^,^^9^^,^^10^ Small traumas suffered by feet that have abnormal (decreased) sensation can cause ulcerations that do not heal primarily.^11^

Such wounds are considered to be complex and are best treated surgically, including debridement of necrotic tissues. This provides preparation for the wound bed, for subsequent skin replacement by means of skin grafts or flaps.^12^

The concept of wound bed preparation was introduced in 2002 by Schultz et al.^13^ The aim was to create favorable conditions that would speed up the endogenous cure of the wound. More recently, wound bed preparation has been used to improve the acceptance of skin grafts.^12^ The preparation includes controlling the microorganisms, reducing the exudate volume and stimulating the granulation tissue.^14^

Nonetheless, in many complex wounds, the conventional dressings that are used to prepare the wound bed still take a long time to achieve ideal conditions. Some alternatives have been tried out over the last 10 years.^15^

The use of negative pressure was proposed by Argenta et al.^16^ and Morykwas et al.^17^ in 1997. This consisted of developing a mechanical system (vacuum-assisted closure) to help the healing process. Negative pressure is created by a machine that is connected by a plastic tube to a sponge placed over the wound bed. The pressure is adjusted to between -50 and -125 mmHg, continuously or intermittently. The wound bed should be completely covered by the sponge, thus creating an environment under vacuum when the machine is switched on.

We introduced this method to Hospital das Clínicas, Faculdade de Medicina da Universidade de São Paulo (HCFMUSP) in 2002, to treat complex wounds. An initial paper reported three cases of wounds for which the vacuum system was used.^18^ A more extensive report on our clinical experience with the vacuum system, mostly on pressure sores, was presented by our group in 2006.^19^

The purpose of the present paper is to present a series of 84 cases of negative pressure therapy on lower-limb wounds among diabetic patients.

## CASE SERIES

Eight-four diabetic patients with a single complex wound on one lower limb were treated using negative pressure (vacuum system) at the Plastic Surgery Division of HCFMUSP, between 2004 and 2006.

All the patients were informed about the proposed treatment and consented to the use of this treatment. This study was approved by the Ethics Committee of HCFMUSP.

The patients were first seen by the vascular surgery service or were referred by their primary physician for a consultation with our complex wound group of the Plastic Surgery Division. All the patients underwent Doppler examination regarding the vascular status of their legs and were considered satisfactory. There was no indication for revascularization or amputation of the limb in any case. The patients’ diabetes status was controlled by their primary physician and was not part of this study.

In most cases, the ulcer had been present for a time of between two and three months. It had been treated by means of conventional dressings alone, while awaiting spontaneous closure.

Surgical debridement was usually indicated, if needed, and any necrotic tissue was removed as soon as possible, as dictated by the patient’s general condition. After removal of the necrosis, a less-than-ideal wound bed was almost always revealed. The quality of the wound bed was assessed in terms of the poor vascularity, gross inflammatory reaction or signs of infection that left doubts regarding whether the “take” of the skin graft would be assured.

The vacuum system (V.A.C.® Vacuum Assisted Closure, KCI Inc., San Antonio, Texas, USA) was then installed. The sponge was placed directly on the wound, and the connections with the machine enabled a pressure of minus 125 mmHg. Sponge shrinkage within the sealed environment indicated that the machine was effective. The sponge was changed every 72 hours, and the condition of the wound bed was reassessed at that time.

The wound bed was considered prepared, i.e. ready for surgical closure (mostly by means of a meshed skin graft, but sometimes by flaps), when the volume of exudate was less than 20 ml/day, no signs of infectious process were seen around the wound and healthy granulation tissue could be observed over at least 75% of the wound surface. The time elapsed between installation of the vacuum machine at the first consultation and the reconstruction (wound preparation) was recorded, along with the outcome after the plastic surgery.

Skin grafts were indicated as the first choice for diabetic ulcers. Local flaps were used on smaller wounds or when specialized structures like tendons or bones were exposed and needed a thicker composite tissue. In some special indications, a microvascular muscle-free flap had to be transferred.

**Figure 1.**
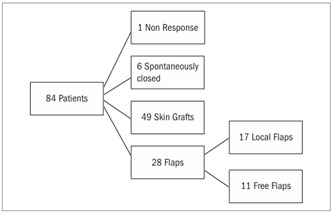
Distribution of the patients according to the treatment chosen.

For 65 (77.3%) of the patients, the mean time taken to prepare the wound was 7.51 ± 1.87 days. For another 12 patients (14.2%), it was 10.87 ± 1.48 days and for one patient (1.3%), the negative pressure therapy was not efficient for improving the quality of the wound bed. For six patients (7.14%), the wound closed in nine days without the need for surgical reconstruction: only the vacuum system was used (Figure 1).

**Figure 2.**
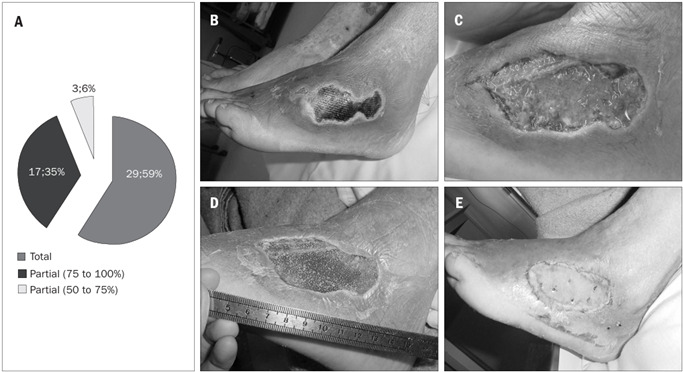
Results from skin grafts. A - Results from patients undergoing skin graft surgery. B - Wound on dorsum of foot before surgical debridement. C - Wound after surgical debridement. D - Wound after seven days of negative pressure therapy. E - Wound after skin grafting.

For 49 patients (58.4%), the wound was successfully closed by means of partial-thickness skin grafts taken from the thigh area. Most of the grafts were meshed (using a mesh graft machine) (Zimmer® Inc., Dover, Ohio, USA) and the vacuum system was applied over the graft, in order to enhance the integration of the skin grafting (Figure 2).

For 28 patients (33.4%), skin or muscle-skin flaps were the method chosen for closing the wound, usually because there was some exposed structure (bone or tendon) that would be better treated by flap coverage.

**Figure 3.**
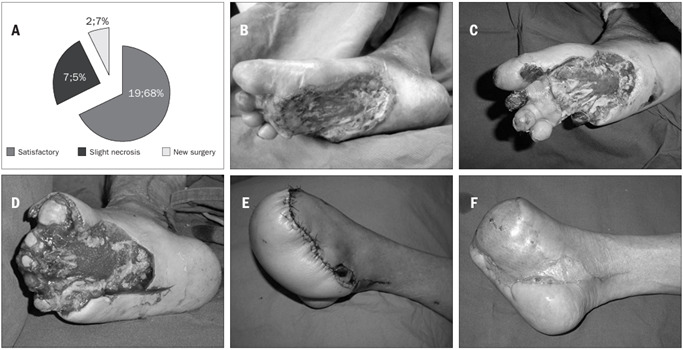
Results from treatment with flaps. A - Distribution of patients undergoing skin flap surgery. B - Wound on foot before surgical debridement. C - Appearance of the wound after first debridement and seven days of negative pressure therapy. Amputation of the necrotic toes was indicated. D - Wound located on the sole of the same diabetic patient after second surgical debridement and amputation of the toes. The heads of the metatarsal bones can be seen. E - Anterolateral microsurgical flap from thigh. F - Result after six months of defatting of the flap.

Nineteen (22.6%) cases presented a satisfactory outcome, with no necrosis or dehiscence of the flap, and nine (10.7%) presented small necrotic areas that healed after some short delay (Figure 3). No complications relating to the use of the vacuum device were observed.

## DISCUSSION

For proper resolution of any chronic wound, a logical sequence of events should be followed in order to transform the wound bed into a healthier condition and then achieve healing. This may take place using intrinsic mechanisms alone, or it may be helped by autogenous tissue transplantation, skin grafts or flaps. These sequential events consist of debridement (removal of necrotic tissue), edema control, reduction of exudate, reduction of the bacteria count and increasing the numbers of blood vessels, with correspondingly better granulation tissue.^13^

The negative pressure action elicited by the vacuum system has been associated with controlling the exudate, reducing the bacterial population and stimulating the formation of granulation tissue since the first patient with a complex wound was treated by Argenta et al.,^16^ 16 years ago.

These effects were clearly demonstrated in the present series. They led to improvements in the treatment of the diabetic ulcer, over a shorter time. Although we did not conduct any comparisons with controls, it is well known from practical experience of any surgery procedure that, with conventional dressings, much more time is needed to prepare a wound bed. Furthermore, although we did not investigate the costs, the reduction in the time taken to close the wound will have had a positive impact regarding reduction of the cost of the treatment.

The mechanism through which the negative pressure achieved the increase in the blood vessel count, i.e. an angiogenic effect, is not completely clear yet. The vacuum system promotes the removal of excessive fluids in the wound, thus reducing the bacterial population and the edema. There is consequently an improvement in the blood flow to the area, thereby leading to better-quality granulation tissue.^20^^,^^21^ The mechanical force applied to the wound bed by the vacuum may explain the cell proliferation, since this would act as a physical substitute for the normal extracellular matrix. This is important for restarting the proliferation phase of wound healing.^22^^,^^23^ Another possible explanation for the action of the vacuum system could be that it removes proteolytic enzymes, especially the metalloproteinases and cytokines in the exudate. These enzymes degrade the extracellular matrix and prevent wound resolution.^24^

In an experimental study on pigs, Morykwas et al.^17^ showed that the blood flow around the wound increased gradually with each elevation of 25 mmHg of vacuum applied to the wound. The improved blood flow was optimized at -125 mmHg. On the other hand, when the vacuum was greater than -400 mmHg, the blood flow fell back to below the baseline.

The vacuum system has also been shown to enhance the quality of the granulation tissue in the wound. Granulation tissue is a mixture of blood vessels and connective tissue and it plays an essential role in cell growth in the wound and thus in its closure. It is considered, clinically, to have a good appearance when it presents a bright red color, a “beefy” appearance.

In the same experimental study,^17^ two groups of wounds were compared: one using the vacuum system and the other covered by saline dressings alone. It was observed that there was a significantly higher growth rate for the granulation tissue in the vacuum group. Moreover, intermittent negative pressure at -125 mmHg seemed to be more efficient than continuous application was. Chen et al.^25^ showed experimentally that negative pressure caused an increase in the number of capillary blood vessels that sprouted, as measured by biopsies and compared with controls.

This angiogenic effect from negative pressure therapy seems to be a very important factor in reducing the time needed for wound bed preparation and thus for the wound to be able to receive and integrate skin grafts. In cases of diabetic foot ulcer, as observed in the present series, this effect will correspond to a reduction in the duration of hospitalization and the need for antibiotics.

The decrease in the bacterial population, down to under the level of 105 colonies in the tissue cultures, will have been important not only because it reduced the expenditure on antibiotics, but also because it enhanced the oxygen and nutrient levels. These are important as healing mechanisms and would have been diverted for the needs of the bacteria. So far, no consensus has been reached regarding the role of negative pressure therapy on bacteria clearance from the wound, although Morykwas et al.^17^ showed that there was a decrease in bacterial counts in the vacuum group, compared with controls.

In our hospital, quantitative counting of tissue bacteria is not done routinely and for this reason, we cannot affirm that the vacuum therapy applied to the wound had the effect of decreasing the bacterial count. Nonetheless, the clinical appearance of the wound, the quality of the exudate and even the smell of the wound pointed towards diminished colonization among most of the diabetic patients that we treated.

The low percentage of skin graft losses also points towards this effect of infection control in the wound. It is well known that skin grafts do not incorporate when the wound is massively infected.^26^ In most cases here, antibiotics were not prescribed during the vacuum system treatment, which usually lasted for one to two weeks.

Contraction of the wound bed was observed. It is well known that this is one the natural mechanisms of reduction of the size of the wound and promotion of primary healing. In our series, contraction was not important because we performed operations on our patients and covered the wounds with grafts or flaps, with the exception of six cases. The negative effect of wound contraction was prevented by the tissue transfer.

Our previous clinical experience with the treatment of diabetic ulcers has shown that, if clinical dressings alone are used, it has to be expected that a long time will be required (usually more than two months) for the wound bed to reaches a good condition for accepting skin grafts.

## CONCLUSIONS

In this series of patients, we observed that negative pressure therapy had a positive effect on the treatment of complex wounds among diabetics. It enabled wound bed preparation in only a short time, and allowed us to successfully close the wound using skin transplants. It is possible that the duration of hospitalization and the overall treatment time might be shorter than with conventional dressing, but further randomized clinical trials are still needed to confirm this hypothesis.
